# Effect of lentivirus-mediated growth and differentiation factor-5 transfection on differentiation of rabbit nucleus pulposus mesenchymal stem cells

**DOI:** 10.1186/s40001-021-00624-5

**Published:** 2022-01-13

**Authors:** Kun Zhu, Rui Zhao, Yuchen Ye, Gang Xu, Changchun Zhang

**Affiliations:** 1 Department of Orthopaedics, The First Affiliated Hospital of Bengbu Medical College, No. 287, Changhuai Road, Bengbu, 233000 Anhui China; 2 Department of General Medicine, Bengbu Medical College, Bengbu, China

**Keywords:** Nucleus pulposus mesenchymal stem cells, Lentivirus, Transfection, Growth and differentiation factor-5, Intervertebral disc degeneration

## Abstract

**Background:**

Intervertebral disc degeneration (IDD) is a natural progression of age-related processes. Associated with IDD, degenerative disc disease (DDD) is a pathologic condition implicated as a major cause of chronic lower back pain, which can have a severe impact on the quality of life of patients. As degeneration progression is associated with elevated levels of inflammatory cytokines, enhanced aggrecan and collagen degradation, and changes in the disc cell phenotype. The purpose of this study was to investigate the biological and cytological characteristics of rabbit nucleus pulposus mesenchymal stem cells (NPMSCs)—a key factor in IDD—and to determine the effect of the growth and differentiation factor-5 (GDF5) on the differentiation of rabbit NPMSCs transduced with a lentivirus vector.

**Methods:**

An in vitro culture model of rabbit NPMSCs was established and NPMSCs were identified by flow cytometry (FCM) and quantitative real-time PCR (qRT-PCR). Subsequently, NPMSCs were randomly divided into three groups: a transfection group (the lentiviral vector carrying GDF5 gene used to transfect NPMSCs); a control virus group (the NPMSCs transfected with an ordinary lentiviral vector); and a normal group (the NPMSCs alone). FCM, qRT-PCR, and western blot (WB) were used to detect the changes in NPMSCs.

**Results:**

The GDF5-transfected NPMSCs displayed an elongated shape, with decreased cell density, and significantly increased GDF5 positivity rate in the transfected group compared to the other two groups (*P* < 0.01). The mRNA levels of Krt8, Krt18, and Krt19 in the transfected group were significantly higher in comparison with the other two groups (*P* < 0.01), and the WB results were consistent with that of qRT-PCR.

**Conclusions:**

GDF5 could induce the differentiation of NPMSCs. The lentiviral vector carrying the GDF5 gene could be integrated into the chromosome genome of NPMSCs and promoted differentiation of NPMSCs into nucleus pulposus cells. Our findings advance the development of feasible and effective therapies for IDD.

**Supplementary Information:**

The online version contains supplementary material available at 10.1186/s40001-021-00624-5.

## Background

Degenerative disc disease (DDD) is a pathological condition recognized as a major contributor to chronic lower back pain (LBP) with a severe impact on the quality of life of patients. It is associated with intervertebral disc degeneration (IDD) which is a natural progression of age-related processes. The intervertebral disc (IVD) is an important component of the spinal column enabling blending, flexion, and torsion of the spine. It is composed of a proteoglycan-rich nucleus pulposus (NP), which is constrained by the surrounding annulus fibrosus and the cartilaginous endplates. Apoptosis of the nucleus pulposus cells (NPCs) and a decrease in cell viability are widely recognized as key factors for disc degeneration, leading to a decrease in extracellular matrix syntheses such as collagen type II and glycoprotein [1, 2]. As a promising treatment approach for DDD, therapies are aimed at the prevention or reversal of IDD.

Nevertheless, current conservative and invasive interventions for IDD aimed at improving LBP are mainly focused on alleviating the symptoms rather than restoring the disc structure and biomechanical function of IDD. Alternatively, the number of NPCs could be supplemented via artificial intervention to potentially inhibit or reverse IDD. For instance, targeting nucleus pulposus mesenchymal stem cells (NPMSCs), a type of stem cell in the NP with strong proliferation and differentiation capabilities, could be a promising therapy for IDD [3, 4].

Current studies have shown that GDF5 can repair degenerative intervertebral discs and promote proteoglycan and collagen type II protein levels [5, 6]. Furthermore, the GDF5 gene has been transfected into human-induced pluripotent stem cells to promote disc regeneration in rats [7]. Despite limited research, biological intervention could be a promising treatment for DDD that could impact future management of LBP. This study investigated the effect of GDF5 transfection on the differentiation of rabbit NPMSCs. It is expected that the results of this study will lay the experimental foundation for the development of a new functional vector material combined with GDF5 transfection to prevent and treat IDD with NPMSCs.

## Materials and methods

Healthy adult rabbits were purchased from the Experimental Animal Feeding and Management Center of Bengbu Medical College, China.

### Main materials and instruments

Cells: rabbit NPMSCs were isolated and cultured in our laboratory.

Instruments: ultra-clean working table (HVAC Purification Equipment, Bengbu, China), cell culture box (Thermo Fisher Scientific, Waltham, MA, USA), micro-centrifuge (Allsheng Instruments, Hangzhou, China), PCR instrument (Lattice Scientific Instrument, Hangzhou, China), and flow cytometer (Becton Dickinson, Franklin Lakes, NJ, USA).

Reagents: DMEM (C11995500CP; Gibco, Billings, MT, USA), RPMI 1640 (C11875500BT; Gibco) fetal bovine serum (#04-002-1a; BioIND, Beit-Haemek, Israel), antibiotic–antimycotic (#15240-112; Life Technologies, Carlsbad, CA, USA); PBS, pH 7.4 (#10010-023; Gibco); Trypsin–EDTA (0.05%) (#25300-054; Life Technologies), bovine serum albumin (#15561012; Life Technologies), Lipofectamine® 2000 transfection reagent (#11668-019; Life Technologies), Opti-mem® I reduced serum medium (#31985-062; Life Technologies); western blot (WB) and IP cell lysis solution, predye protein marker (Fermentas, Vilnius, Lithuania); and polyvinylidene membrane (Millipore, Burlington, MA, USA).

Antibodies: CD90 antibody (ab225; Abcam, Cambridge, UK), CD105 antibody (ab221675; Abcam), CD34 antibody (bs-0646R; Bioss, Woburn, MA, USA), CD45 antibody (bs-0522R; Bioss), FITC-labeled sheep anti-mouse IgG (bs-0296g-fitc; Bioss), FITC-labeled sheep anti-rabbit IgG (bs-0295g-fitc; Bioss), GDF5 antibody (ab93855; Abcam), and anti-actin antibody (SAB4200248; Sigma-Aldrich, Burlington, MA, USA).

Kits: Ultrapure RNA extraction kit (CW0581S; Kangwei Century Biotechnology, Beijing, China), SuperRT cDNA synthesis kit (CW0741S; Kangwei Century Biotechnology), UltraSYBR Mixture (High ROX, CW2602M; Kangwei Century Biotechnology), and BCA protein quantitative kit (Beyotime Biotechnology, Shanghai, China).

### Cell isolation culture and identification

Acquisition of NPMSCs: briefly, the rabbit NP tissues were harvested, digested by collagenase, centrifuged, resuspended, and the NPCs were obtained. Then the suspension of NPCs was centrifuged. Subsequently, the supernatant fluid was removed, and the complete medium of mesenchymal stem cells was added. Finally, the suspension was filtered to obtain NPMSCs. When the cell fusion rate reached 80–90%, they were digested by trypsin and re-inoculated for the subculture of the NPMSCs. The morphological and biological characteristics of the cells were observed under an inverted microscope.

The surface immunophenotype of the NPMSCs in rabbits was identified by flow cytometry (FCM). The third generation of NPMSCs cells was digested with trypsin and then centrifuged. Subsequently, the precipitated cells were collected, washed with PBS, and diluted to the cell suspension. Anti-rabbit CD90, CD105, CD34, and CD45 antibodies were added, respectively. The suspension was incubated at 25 °C, washed with PBS, incubated with FITC-labeled sheep anti-mouse/sheep anti-rabbit IgG secondary antibody (in the absence of light), and washed with PBS. After the mixing procedure, the mixture was detected by FCM.

Quantitative real-time PCR (qRT-PCR): samples were collected to extract the RNA, and the mRNA level of the target gene was detected by qRT-PCR. RNA was extracted from the NPMSCs, and then the cDNA was synthesized. Subsequently, qRT-PCR was performed, using the GAPDH gene as an internal reference, and the experiment was performed in triplicate for each of the genes per sample. After adding each component to the PCR tube, we carefully sealed the plate membrane, mixed it evenly, and centrifuged the solution to generate a pellet at the bottom of the tube. PCR amplification conditions were based on the instructions of the qRT-PCR kit. According to the instructions of the instrument, the PCR experiment was performed three times. The data were collected and analyzed.

### Cell transfection and observation

NPMSCs were divided into three groups: a transfection group (lentiviral vector carrying the GDF5 gene used to transfect NPMSCs); a control virus group (NPMSCs transfected with an ordinary lentiviral vector); and a normal group (the NPMSCs alone).

Transfection methods: the lentivirus plasmids were diluted to 25 L of Opti-MEM® I reduced serum medium, and 0.5 L of Lipofectamine 2000 was diluted to 25 L of Opti-MEM® I reduced serum medium. The solutions were gently mixed, followed by a period of keeping them static, before adding the mixtures to the three groups for culturing.

The gene transfection rate was identified by FCM. All cell groups were digested with trypsin and centrifuged. The precipitated cells were collected followed by washing with PBS and diluting the cell suspension. Anti-rabbit GDF5 was added and incubated for 30 min. The cells were incubated with FITC-labeled sheep anti-rabbit IgG secondary antibody and washed with PBS. Following mixing, the positivity rate of GDF5 in each group was determined by FCM.

Cell counting: Kit-8 (CCK8; Invitrogen, Waltham, MA, USA) was used to measure cell proliferation on days 1, 4, and 7, respectively. The absorbance of the solutions was measured at 450 nm using a microplate reader (BioTek, Winooski, VT, USA).

qRT-PCR was used to detect the mRNA levels of keratin 8, 18, 19 (Krt8, Krt18, and Krt19) in all groups. Cell samples were collected, followed by RNA extraction, and finally, cDNA synthesis was performed. The procedures were identical to those described previously.

The protein levels of KRT8, KRT18, and KRT19 in all groups were detected by WB. The steps include the extraction of the total protein of cells, quantification of protein, protein electrophoresis, film transferring, sealing and incubation, PVDF membrane chemiluminescence, followed by developing and fixing. Finally, the image analysis and statistics were performed: the developing strip was analyzed, and the optical density was scanned by Image J v1.8.0. The relative expression was calculated as the ratio between the optical density of the target band and the corresponding value of the β-actin.

### Statistical methods

All the experiments were performed in triplicate for each of the genes per sample. The mean values obtained were compared by analysis of variance (ANOVA). Data were presented as mean ± standard deviation. One-way ANOVA was used for comparisons between the groups, and independent sample t-tests were used for pairwise comparisons. Differences were statistically significant at *P* < 0.05.

## Results

### Culture and identification of NPMSCs

**Fig. 1:**
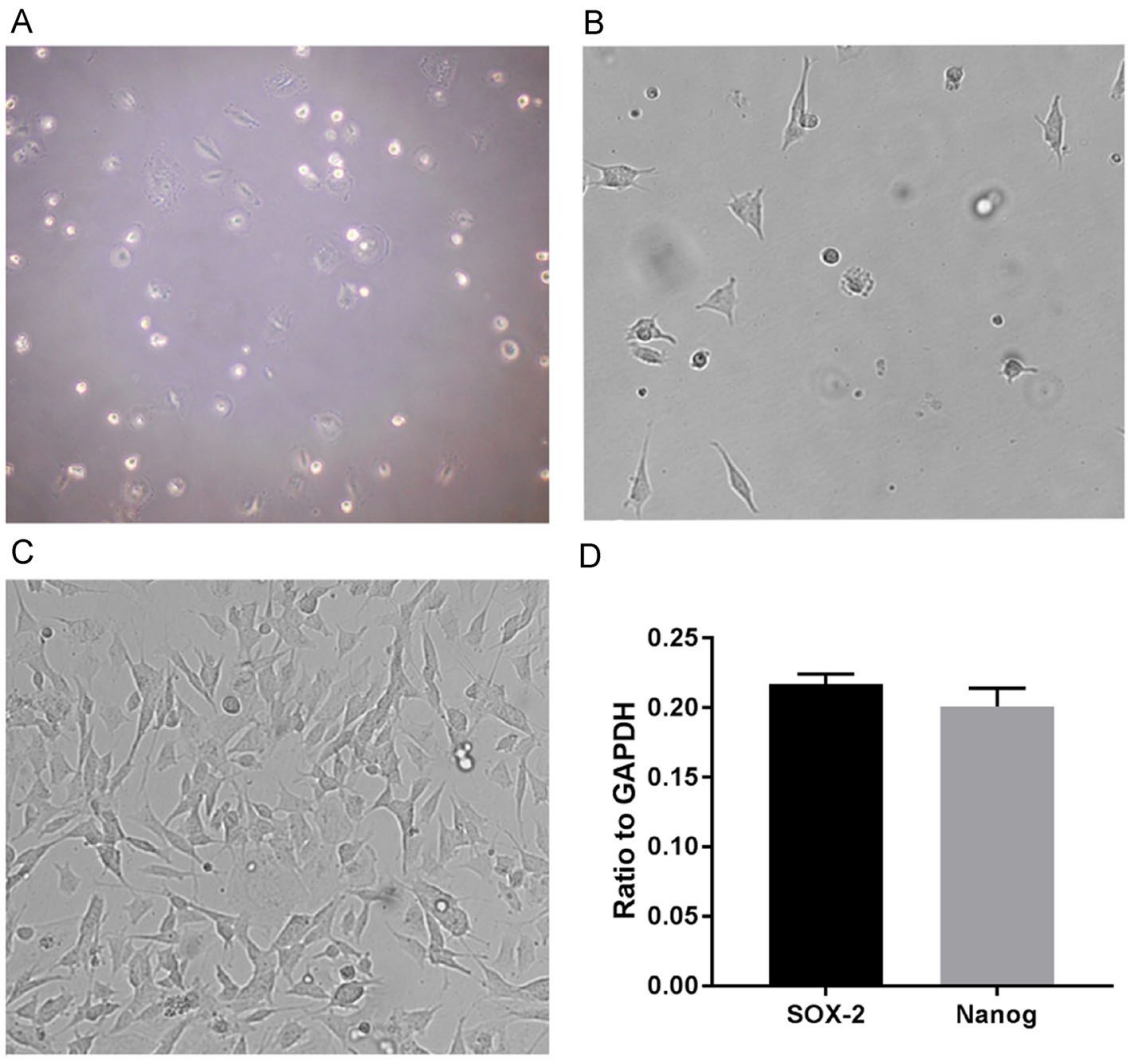
Primary, passaged, and transfected NPMSCs. **A** Completely digested NPMSCs (magnification, × 200); **B** primary NPMSCs (magnification, × 200); **C** third generation (magnification, × 200); **D** qRT-PCR results: NPMSCs could express the stem cell genes SOX2 and Nanog

Most of the NPMSCs screened by the complete culture medium of the mesenchymal stem cells (MSCs) were single cells after digestion (Fig. 1A); some of the cells adhered to the wall of culture flask after primary culture for 4 × 6 days, and the morphology of the cells were spindle-shaped or polygonal (Fig. 1B). The cell colony formation was observed after 12 days and the cell fusion rate reached 90% after 3–4 weeks. After the passage, the cell proliferation increased significantly with the following passages taking only ~ 1 week, and the number of spindle cells began to increase. After passaging to the third generation, most of the cells were fusiform (Fig. 1C).

**Fig. 2:**
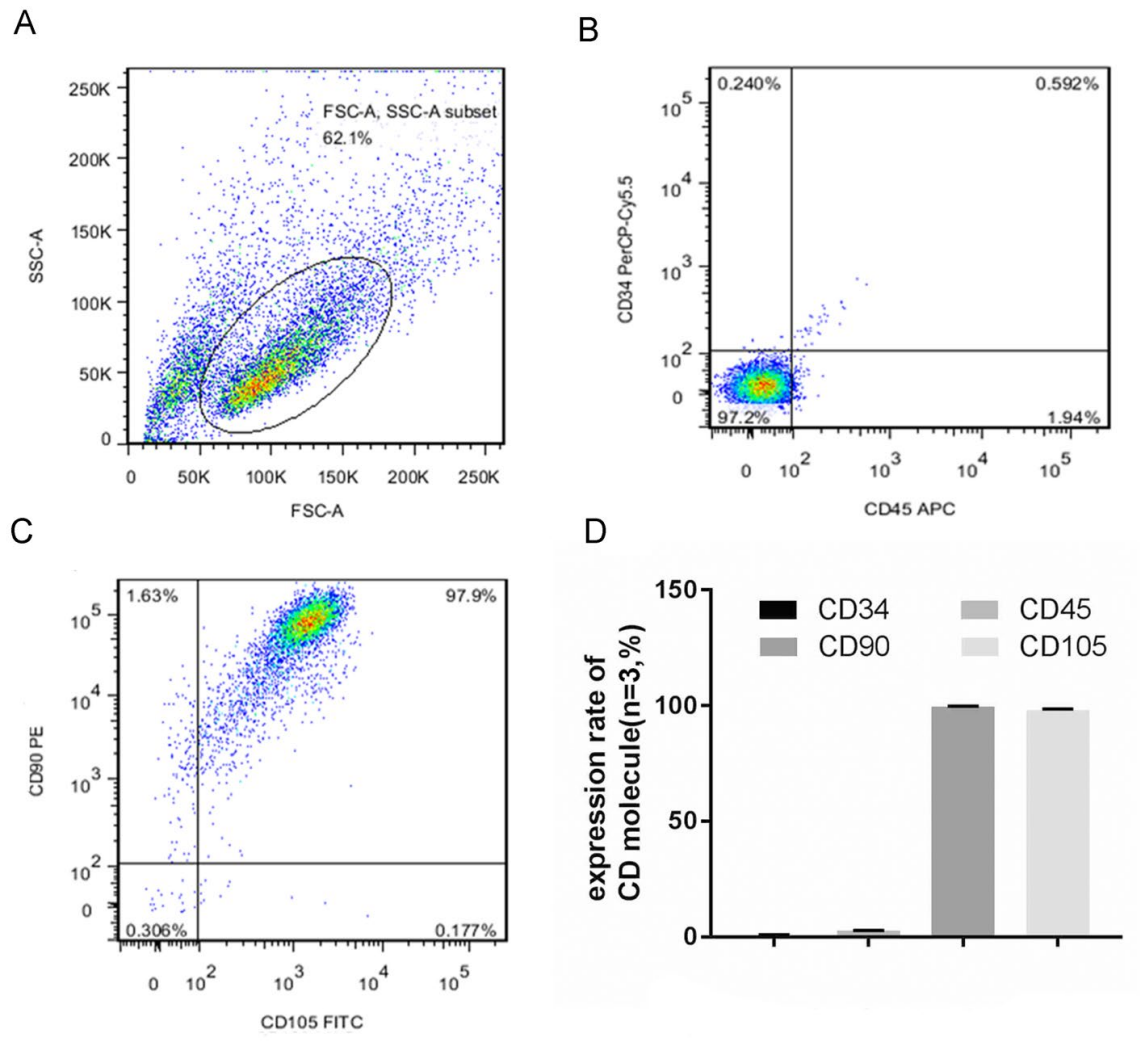
Identification of NPMSCs by FCM. **A** Cell fragments removed by FSC/SSC scatter plot, and NPMSCs indicated in the circle. **B** The negative immunophenotypes CD34 and CD45 of NPMSCs. **C** The positive immunophenotypes CD90 and CD105 of NPMSCs. **D** The expression rate of the surface immunophenotype CD molecules of NPMSCs

The mesenchymal stem cell markers CD34 and CD45 were negatively expressed in FCM (Fig. 2B), whereas CD90 and CD105 were positively expressed (Fig. 2C). The expression rate of the surface immunophenotype CD molecules of NPMSCs is shown in Fig. 2D.

qRT-PCR results: the NPMSCs expressed the stem cell genes SOX2 and Nanog (Fig. 1D).

### Detection of GDF5 gene-transfected NPMSCs

NPMSCs displayed spindle-shaped morphology before transfection (Fig. 4A). After transfection, the shape of the NPMSCs elongated and the cells density decreased (Fig. 4B). Next, we studied the effects of transfection on cell proliferation. The results of the CCK8 method are shown in Fig. 4. The number of cells per group continued to increase during the experiment. On the 4th and 7th days of culture, cells in the transfected group showed better viability, and the cell proliferation rate was significantly higher than that in the untransfected group. Thus, these results show that the proliferation of cells in the transfected group was improved (Fig. 4C).

**Fig. 3:**
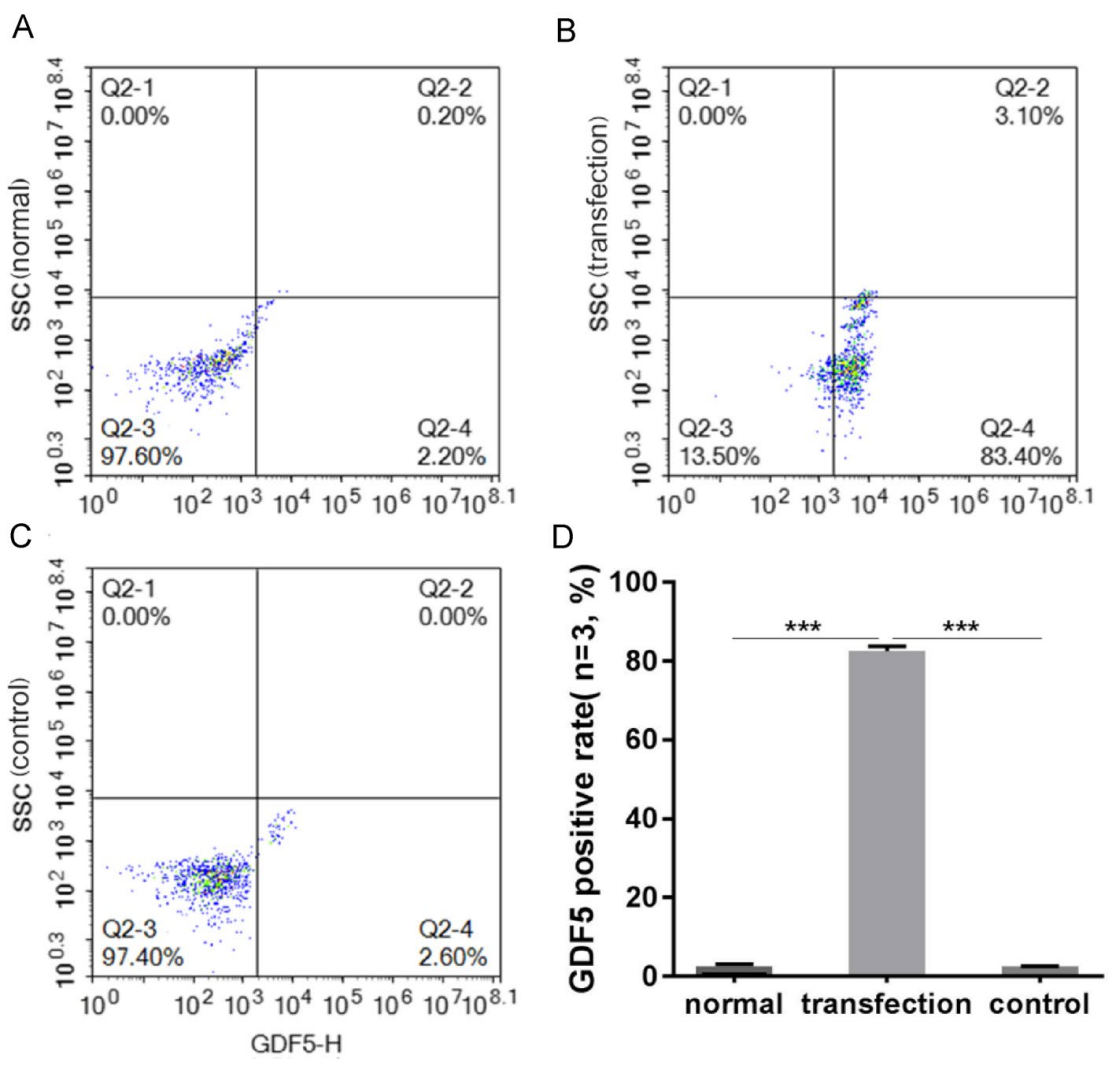
FCM cell transfection rate. **A** GDF5 positive rate (%) in the normal group; **B** GDF5 positive rate (%) in the transfection group; **C** GDF5 positive rate (%) of the control group; **D** The GDF5 positive rate (%) of the transfection group was significantly higher than that of either the normal or control groups (*P* < 0.01)

FCM showed that the GDF5-positive expression rate (%) of the transfected group was significantly higher than that of either the normal group or the control virus group (*P* < 0.01) (Fig. 3D). The positive rates (%) of GDF5 expression of the three groups are shown in Fig. 3A–C.

**Fig. 4:**
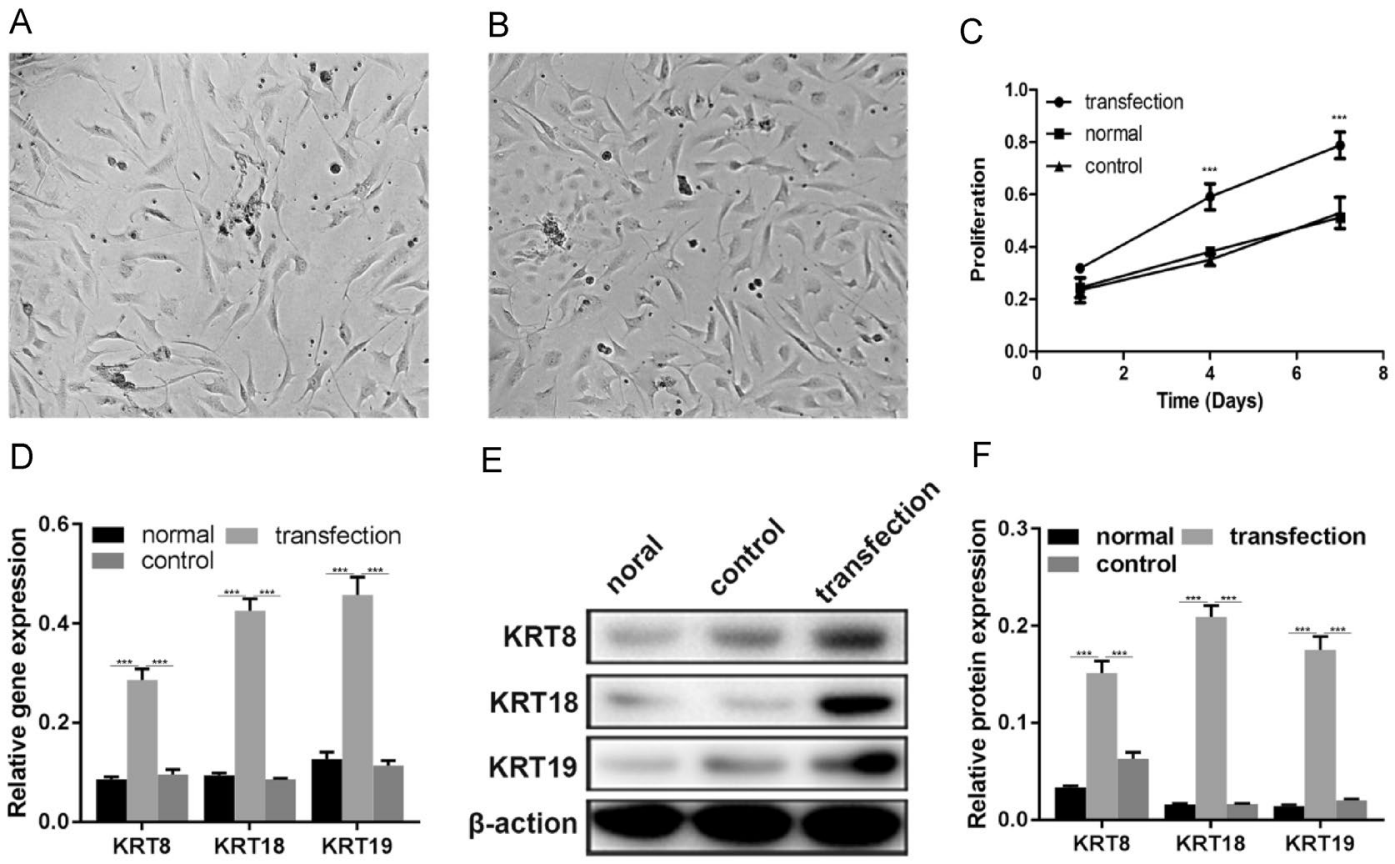
**A** NPMSCs before transfection (magnification, × 200); **B** Recombinant NPMSCs after transfection (magnification, × 200); **C** Cell proliferation of NPMSCs in three groups at days 1, 4, and 7, respectively (*P* < 0.01); **D** qRT-PCR results: mRNA levels of KRT8, KRT18, and KRT19 significantly increased in the transfection group compared to the other two groups (*P* < 0.01). **E** WB detected the protein levels of KRT8, KRT19, and KRT19 in the three groups. **F** WB results: protein levels of KRT8, KRT18, and KRT19 significantly increased in the transfection group compared to the other two groups (*P* < 0.01)

The qRT-PCR analysis in the three groups showed that mRNA expression levels of KRT8, KRT18, and KRT19 in the transfected group were significantly higher than that in the other two groups (*P* < 0.01) (Fig. 4D).

The WB analysis of the three groups showed that the protein levels of KRT8, KRT18, and KRT19 in the transfected group significantly increased compared to the other two groups (*P* < 0.01) (Fig. 4E–F).

## Discussion

Lumbar degenerative disease, caused by lumbar disc degeneration, severely affects the quality of life of patients and has become a serious social problem. Unfortunately, current treatment approaches including invasive and conservative interventions cannot fundamentally reverse disc degeneration [8, 9]. However, as research into the molecular mechanisms and genetic engineering of disc degeneration increase, IDD may be inhibited or reversed at the gene level.

MSCs are pluripotent stem cells originating from the mesoderm, which can differentiate into bone, cartilage, fat, muscle, ligament, tendon, and other tissues [10, 11]. In 2006, the International Association for Cell Therapy [12] proposed an acknowledged standard for the definition of MSCs as follows: (A) for in vitro culture, cells grow adherently to the wall of culture flask; (B) some specific antigens (markers) are expressed on the cell surface; and (C) the cells possess the ability to differentiate into adipocytes, osteoblasts, and chondrocytes. Many studies have shown that there is a specific type of cell in the intervertebral disc tissue with the ability to differentiate into osteoblasts, chondroblasts, and lipid cells. Additionally, this cell also characteristically expresses the surface protein molecules of MSCs and can complete the three-line induced differentiation. These types of cells have been identified as NPMSCs [13–16].

NPMSCs are the precursor of NPCs, which exist in healthy and degenerative IVD tissues, and has the potential to proliferate and differentiate into NPCs [17]. Furthermore, these cells show great potential for application in regenerative and repairing sciences [3, 4]. Li et al. successfully isolated human NPMSCs by fluorescence-activated cell sorting and showed that the cells specifically expressed tyrosine kinase receptor 2 and ganglioside 2. The cells had significant proliferation and differentiation potential and could differentiate into osteoblasts, adipocytes, and chondrocytes [18]. Erwin et al. demonstrated that NPMSCs possessed a powerful capability to differentiate and proliferate in animal studies, and played an important role in IVD repair, nerve repair, and other renewable medical applications [19].

Lin et al. cultured NPMSCs of rats in vitro to induce differentiation and found that NPMSCs could express the stem cell genes SOX2, Oct4, and Nanog [20]. Zhang et al. compared rat NPMSCs and bone marrow MSCs in vitro and observed that both of them could differentiate into bone, cartilage, and fat, while also expressing the stem cell genes SOX2, Oct4, and Nanog [21]. In this study, FCM was used to detect the surface immunophenotype CD molecules of NPMSCs. We found that the immunophenotypes CD90 and CD105 were positive, while CD34 and CD45 were negative. Furthermore, qRT-PCR showed positive expression of the stem cell genes SOX2 and Nanog, in line with previous publications.

Researchers have found that human notochord cells (NCs), later identified as NPMSCs, gradually disappear with age-related disc degeneration [22]. Rodrigues-pinto et al. found that KRT8, KRT18, and KRT19 were specific markers of human NCs, and were expressed at all stages of NCs [23]. Minogue et al. detected the mRNA levels of KRT8, KRT18, and KRT19 in bovine NPCs and NCs by RT-PCR, and found that both of them expressed KRT8, KRT18, and KRT19, though slightly higher in NCs compared to NPCs [24]. Meanwhile, other researchers have found that NPCs express KRT8, KRT18, KRT19, and other gene phenotypes; however, their expression levels were higher in NPCs than that of articular chondrocytes and ring fibroblasts [25, 26].

GDF5, also known as BMP14, is a member of the bone morphogenetic protein (BMP) family. BMP was originally thought to be a component of the mineralized bone matrix, which can induce the formation of new bone tissue when fractures or ectopic ossification occur [27, 28]. Current studies have shown that GDF5 can repair degenerative intervertebral discs and promote proteoglycan and collagen type II protein levels [5, 6]. GDF5 has also been transfected into human-induced pluripotent stem cells to promote disc regeneration in rats [7]. Animal studies have found that the central region of the intervertebral disc of GDF5 knockout mice showed low signal with MRI T2-weighted imaging and the normal lamellar structure of the fibrous ring disappeared. This resulted in atrophy and disorder of the NP tissues, while the content of proteoglycan decreased significantly. The expression of proteoglycan and collagen type II mRNA also decreased, indicating that the deletion of the GDF5 gene was closely related to IDD. Meanwhile, GDF5 also promoted the differentiation of stem cells [29]. In the current study, mRNA and protein levels of KRT8, KRT18, and KRT19 in the transfected group were significantly increased compared with the other two groups, suggesting that the GDF5 gene could promote the differentiation of NPMSCs (see Additional file 1).

Regarding the mechanism of GDF5 acting on NPMSCs, Liu et al. found that GDF5 could inhibit the transcription and expression of the RNA fragment microRNA-34a, reduce the generation of IL-1β, as well as increase the expression of proteoglycan and collagen type II. These findings indicated that GDF5 could delay or stop the degeneration of the disc [30].

There are some limitations to this study. First, the expression of the extracellular matrix in NPCs is a complex process. In this study, only KRT8, KRT19, and KRT19 were detected as representative markers of NPCs. In addition, the current experiment on rabbits cannot fully simulate human disc repair. Future studies should include human cells to further examine cell differentiation and biosynthesis.

## Conclusion

In this study, an in vitro culture model of NPMSCs was established, and techniques such as gene transfection, FCM, qRT-PCR, and WB were used to demonstrate the existence of NPMSCs in rabbit NP tissue. GDF5 was demonstrated to promote the differentiation of rabbit NPMSCs into NPCs in vitro; however, the exact mechanism could not be identified. Thus, further studies to address this limitation are required in future. Although a significant gap exists between the research findings and clinical practice, our current data help uncover the pathogenesis of IDD to advance the development of feasible and effective therapies for IDD.

## Supplementary Information

**Additional file 1:** **Table S1.** The means and standard deviations for the data ($\overline{{\text{x}}}$±SD).

## Data Availability

All data generated or analyzed during this study are included in this manuscript.

## References

[1] Kermani HR, Hoboubati H, Esmaeili-Mahani S, Asadi-Shekaari M (2014). Induction of intervertebral disc cell apoptosis and degeneration by chronic unpredictable stress. J Neurosurg Spine.

[2] Wang J, Chen H, Cao P, Wu X, Zang F, Shi L, Liang L, Yuan W (2016). Inflammatory cytokines induce caveolin-1/β-catenin signalling in rat nucleus pulposus cell apoptosis through the p38 MAPK pathway. Cell Prolif.

[3] Han B, Wang HC, Li H, Tao YQ, Liang CZ, Li FC, Chen G, Chen QX (2015). Nucleus pulposus mesenchymal stem cells in acidic conditions mimicking degenerative intervertebral discs give better performance than adipose tissue-derived mesenchymal stem cells. Cells Tissues Organs.

[4] Yan J, Yang S, Sun H, Guo D, Wu B, Ji F, Zhou D (2014). Effects of releasing recombinant human growth and differentiation factor-5 from poly (lactic-co-glycolic acid) microspheres for repair of the rat degenerated intervertebral disc. J Biomater Appl.

[5] Clarke LE, McConnell JC, Sherratt MJ, Derby B, Richardson SM, Hoyland JA (2014). Growth differentiation factor 6 and transforming growth factor-beta differentially mediate mesenchymal stem cell differentiation, composition, and micromechanical properties of nucleus pulposus constructs. Arthritis Res Ther.

[6] Hu A, Xing R, Jiang L, Li Z (2020). Thermosensitive hydrogels loaded with human-induced pluripotent stem cells overexpressing growth differentiation factor-5 ameliorate intervertebral disc degeneration in rats. J Biomed Mater Res.

[7] Mehrkens A, Matta A, Karim MZ, Kim S, Fehlings MG, Schaeren S, Mark EW (2017). Notochordal cell-derived conditioned medium protects human nucleus pulposus cells from stress-induced apoptosis. Spine J.

[8] Koppenaal T, Arensman RM, van Dongen JM, Ostelo R, Veenhof C, Kloek CJJ, Pisters MF (2020). Effectiveness and cost-effectiveness of stratified blended physiotherapy in patients with non-specific low back pain: study protocol of a cluster randomized controlled trial. BMC Musculoskelet Disord.

[9] Hu Y, Huang L, Shen M, Liu Y, Xiong L (2019). Pioglitazone protects compression-mediated apoptosis in nucleus pulposus mesenchymal stem cells by suppressing oxidative stress. Oxid Med Cell Longev.

[10] Karamini A, Bakopoulou A, Andreadis D, Gkiouras K, Kritis A (2020). Therapeutic potential of mesenchymal stromal stem cells in rheumatoid arthritis: a systematic review of in vivo studies. Stem Cell Rev Rep.

[11] Sheyn D, Ben-David S, Tawackoli W, Zhou Z, Salehi K, Bez M, De Mel S, Chan V, Roth J, Avalos P, Giaconi JC, Yameen H, Hazanov L, Seliktar D, Li D, Gazit D, Gazit Z (2019). Human iPSCs can be differentiated into notochordal cells that reduce intervertebral disc degeneration in a porcine model. Theranostics.

[12] Nolta JA, Galipeau J, Phinney DG (2020). Improving mesenchymal stem/stromal cell potency and survival: proceedings from the International Society of Cell Therapy (ISCT) MSC preconference held in May 2018, Palais des Congres de Montreal, Organized by the ISCT MSC Scientific Committee. Cytotherapy.

[13] Henry N, Clouet J, Le Bideau J, Le Visage C, Guicheux J (2018). Innovative strategies for intervertebral disc regenerative medicine: from cell therapies to multiscale delivery systems. Biotechnol Adv.

[14] Wang W, Deng G, Qiu Y, Huang X, Xi Y, Yu J, Yang X, Ye X (2018). Transplantation of allogenic nucleus pulposus cells attenuates intervertebral disc degeneration by inhibiting apoptosis and increasing migration. Int J Mol Med.

[15] Qi L, Wang R, Shi Q, Yuan M, Jin M, Li D (2019). Umbilical cord mesenchymal stem cell conditioned medium restored the expression of collagen II and aggrecan in nucleus pulposus mesenchymal stem cells exposed to high glucose. J Bone Miner Metab.

[16] Li XC, Tang Y, Wu JH, Yang PS, Wang DL, Ruan DK (2017). Characteristics and potentials of stem cells derived from human degenerated nucleus pulposus: potential for regeneration of the intervertebral disc. BMC Musculoskelet Disord.

[17] Tian D, Liu J, Chen L, Zhu B, Jing J (2020). The protective effects of PI3K/Akt pathway on human nucleus pulposus mesenchymal stem cells against hypoxia and nutrition deficiency. J Orthop Surg Res.

[18] Erwin WM, Islam D, Eftekarpour E, Inman RD, Karim MZ, Fehlings MG (2013). Intervertebral disc-derived stem cells: implications for regenerative medicine and neural repair. Spine (Phila Pa 1976).

[19] Lin L, Jia Z, Zhao Y, Wu Y, Zhao X, Li Y, Guo Z, Chen J, Cheng S, Wang D, Ruan D (2017). Use of limiting dilution method for isolation of nucleus pulposus mesenchymal stem/progenitor cells and effects of plating density on biological characteristics and plasticity. Biomed Res Int.

[20] Zhang H, Ma X, Zhang L, Guan X, Bai T, Xue C (2015). The ability to form cartilage of NPMSC and BMSC in SD rats. Int J Clin Exp Med.

[21] Wang F, Gao ZX, Cai F, Sinkemani A, Xie ZY, Shi R, Wei JN, Wu XT (2017). Formation, function, and exhaustion of notochordal cytoplasmic vacuoles within intervertebral disc: current understanding and speculation. Oncotarget.

[22] Rodrigues-Pinto R, Berry A, Piper-Hanley K, Hanley N, Richardson SM, Hoyland JA (2016). Spatiotemporal analysis of putative notochordal cell markers reveals CD24 and keratins 8, 18, and 19 as notochord-specific markers during early human intervertebral disc development. J Orthop Res.

[23] Minogue BM, Richardson SM, Zeef LA, Freemont AJ, Hoyland JA (2010). Transcriptional profiling of bovine intervertebral disc cells: implications for identification of normal and degenerate human intervertebral disc cell phenotypes. Arthritis Res Ther.

[24] Richardson SM, Ludwinski FE, Gnanalingham KK, Atkinson RA, Freemont AJ, Hoyland JA (2017). Notochordal and nucleus pulposus marker expression is maintained by sub-populations of adult human nucleus pulposus cells through aging and degeneration. Sci Rep.

[25] Wang Z, Leng J, Zhao Y, Yu D, Xu F, Song Q, Qu Z, Zhuang X, Liu Y (2017). N-cadherin maintains the healthy biology of nucleus pulposus cells under high-magnitude compression. Cell Physiol Biochem.

[26] Thorpe AA, Binch AL, Creemers LB, Sammon C, Le Maitre CL (2016). Nucleus pulposus phenotypic markers to determine stem cell differentiation: fact or fiction?. Oncotarget.

[27] Morimoto T, Kaito T, Matsuo Y, Sugiura T, Kashii M, Makino T, Iwasaki M, Yoshikawa H (2015). The bone morphogenetic protein-2/7 heterodimer is a stronger inducer of bone regeneration than the individual homodimers in a rat spinal fusion model. Spine J.

[28] Itoh F, Watabe T, Miyazono K (2014). Roles of TGF-beta family signals in the fate determination of pluripotent stem cells. Semin Cell Dev Biol.

[29] Feng C, Liu H, Yang Y, Huang B, Zhou Y (2015). Growth and differentiation factor-5 contributes to the structural and functional maintenance of the intervertebral disc. Cell Physiol Biochem.

[30] Liu W, Zhang Y, Feng X, Li S, Gao Y, Wang K, Song Y, Yang S, Tu J, Shao Z, Yang C (2016). Inhibition of microRNA-34a prevents IL-1beta-induced extracellular matrix degradation in nucleus pulposus by increasing GDF5 expression. Exp Biol Med (Maywood).

