# Supplementary material for: Effect of lentivirus-mediated growth and differentiation factor-5 transfection on differentiation of rabbit nucleus pulposus mesenchymal stem cells
Source: Eur J Med Res. 2022 Jan 13;27:5. doi: 10.1186/s40001-021-00624-5 (PMC8756615; doi:10.1186/s40001-021-00624-5)
Supplement: Supplementary file 1 — Additional file 1: Table S1. The means and standard deviations for the data ($\overline{{\text{x}}}$x¯±SD). [file 40001_2021_624_MOESM1_ESM.doc]

Table S1 The means and standard deviations for the data (±SD)

|  | Normal group | Transfection group | Control group | *F* | *P-*value |
| --- | --- | --- | --- | --- | --- |
| FCM | 2.633±0.404 | 82.633±0.929* | 2.667±0.208^#^ | 17936.452 | <0.01 |
| CCK8(day 1) | 0.257±0.006 | 0.270±0.010 | 0.253±0.015 | 1.9091 | 0.23 |
| CCK8(day 4) | 0.347±0.006 | 0.590±0.020* | 0.350±0.017^#^ | 238.955 | <0.01 |
| CCK8(day 7) | 0.437±0.055 | 0.770±0.050* | 0.443±0.047^#^ | 42.077 | <0.01 |
| PCR KRT8 | 0.086±0.005 | 0.286±0.023* | 0.095±0.010^#^ | 173.347 | <0.01 |
| PCR KRT18 | 0.094±0.005 | 0.426±0.024* | 0.086±0.002^#^ | 582.562 | <0.01 |
| PCR KRT19 | 0.126±0.014 | 0.458±0.036* | 0.114±0.010^#^ | 213.233 | <0.01 |
| WB KRT8 | 0.034±0.001 | 0.151±0.008* | 0.060±0.007^#^ | 284.524 | <0.01 |
| WB KRT18 | 0.016±0.001 | 0.209±0.011* | 0.016±0.002^#^ | 897.220 | <0.01 |
| WB KRT19 | 0.013±0.002 | 0181±0.017* | 0.020±0.001^#^ | 290.948 | <0.01 |

*Compared with the normal group (*P* < 0.01), ^#^Compared with the transfection group (*P* < 0.01).
